# Telemedicine Service Experience Questionnaire for Chinese Outpatients: Development and Validation Study

**DOI:** 10.2196/60551

**Published:** 2026-05-21

**Authors:** Peicheng Wang, Kun Zhang, Yanhua Chen, Minan Zhao, Peiyao Li, Yi Kuang, Yanrong Du, Weiguo Zhu, Xiao Long, Leiyu Shi, Jiming Zhu

**Affiliations:** 1 Vanke School of Public Health, Tsinghua University, Beijing, China; 2 Department of Health Policy and Management, Bloomberg School of Public Health, The Johns Hopkins University, Baltimore, MD, United States; 3 International Health Service, Peking Union Medical College of Hospital, Beijing, China; 4 China Medical Board, Dongcheng District, Beijing, 100006, China, +86 13810497965

**Keywords:** telemedicine service, outpatients experience questionnaire, validity and reliability, Chinese outpatients, telemedicine, patient satisfaction, questionnaire, China, outpatient, care, information technology, health care system

## Abstract

**Background:**

Telemedicine has rapidly expanded; however, standardized, telemedicine-specific patient-reported experience measures tailored to outpatient workflows are limited in many settings.

**Objective:**

This study aimed to develop and psychometrically validate the Telemedicine Service Experience Questionnaire (TSEQ) for Chinese outpatients using telemedicine services.

**Methods:**

We conducted a web-based survey among outpatients who completed a telemedicine consultation at Peking Union Medical College Hospital between July 1, 2021, to August 31, 2021, and who had used telemedicine services, using an adapted Chinese Patient Experience Questionnaire that encompasses 15 questions across 4 dimensions, to investigate patients’ telemedicine consultation experiences. Item generation was informed by a literature review, workflow mapping, and expert review. We evaluated the factor structure using exploratory factor analysis and confirmatory factor analysis on the full sample with cross-validation. Reliability was assessed using Cronbach α and item-total correlations.

**Results:**

In total, 3338 participants completed the survey (mean age 45.3, SD 17.8 y; n=2182, 65.4% female participants; n=1827, 54.8% with college education or above). The exploratory factor analysis of the final 14-item scale resulted in 4 factors. After scrutinizing the content, these factors were labeled “Service Efficiency,” “Post-treatment,” “Information Guidance,” and “Humanistic Care,” and they demonstrated good internal consistency (Cronbach α values of 0.876, 0.840, 0.962, and 0.876, respectively). Moreover, as the average variance extracted values were greater than 0.5 and the composite reliability values were greater than 0.7, the TSEQ scale has high convergent validity. Our findings suggest that the psychometric properties of the 14-item TSEQ are valid and reliable for assessing telemedicine service experience among Chinese outpatients.

**Conclusions:**

The TSEQ demonstrates a stable multidomain structure with satisfactory reliability and validity for evaluating outpatient telemedicine service experience in China. The instrument can support routine quality monitoring and guide targeted workflow improvements. Future studies should validate the TSEQ in multisite and postpandemic samples and examine measurement invariance across key subgroups.

## Introduction

With the rapid development of information technology and the popularization of the internet, telemedicine services are becoming an innovative approach in the medical field [1] and are being used in a variety of settings, including psychiatric counseling [2], home health care services [3], intensive care units [4], and emergency stroke care [5], to provide patients with efficient and convenient medical care. In recent years, the worldwide COVID-19 outbreak has accelerated the spread and adoption of telemedicine services in health care [5]. Faced with the constraints of outbreaks and the need for prevention and control, telemedicine services protect patients, clinicians, and communities from exposure. In addition, they provide a platform where physicians and patients can interact using smartphones or webcam-enabled computers, regardless of time or location [6]. Particularly in China, a vast country with rural areas and dispersed populations, telemedicine services hold great potential and advantages in addressing the unequal distribution of health care resources and improving the quality of medical services [7]. The Chinese government has explicitly included telemedicine services as a key area in the development of the national health care system. In 2018, the National Health Commission of China launched the Action Plan for Further Improvement of Medical Services (2018‐2020). The program encourages medical institutions and research institutes to strengthen innovative research and development and vigorously promotes the integration of telemedicine technology with medical resources to facilitate the gradual development of telemedicine [8].

As an emerging health care model, patient satisfaction with telemedicine services is an important indicator of the quality of telemedicine services and an important driving force for the development of telemedicine services. Collecting and analyzing patient feedback can help medical institutions and decision-makers to comprehensively understand patients’ demands and expectations, improve and optimize service quality, attract more patients to accept telemedicine services, and promote their wide application in health care. Studies have shown that the use of telemedicine services will only expand if patients and health care professionals are at least as satisfied with telemedicine services as they are with in-person visits [9]. In addition, highly satisfied patients are likely to have higher medication adherence, leading to better health outcomes [10].

Several studies have been conducted to explore the satisfaction with telemedicine services, such as the Telemedicine Satisfaction and Usefulness Questionnaire [11], Telemedicine Satisfaction Survey [12], and Telehealth Usability Questionnaire [13]. Nevertheless, telemedicine-specific, psychometrically validated patient-reported experience instruments tailored to Chinese outpatient workflows remain limited. In China, the scales currently available for measuring telemedicine service satisfaction are mainly based on the extension and improvement of traditional health care service satisfaction scales and lack sufficient consideration of the specific characteristics and unique features of telemedicine services [14]. For example, these scales may not fully capture telemedicine-specific processes (eg, digital information guidance, postconsultation payment and medication delivery, and privacy or security concerns).

To address this gap, we developed the Telemedicine Service Experience Questionnaire (TSEQ), using various items adapted for patient satisfaction surveys among Chinese outpatients. Our primary objective in this paper was to assess the appropriateness of the TSEQ scale as an instrument to measure patient satisfaction across a broad range of outpatient populations in China. Rather than measuring technology usability alone, the TSEQ is designed to capture the service-process experience across the entire outpatient telemedicine journey, including preconsultation guidance, interaction quality, and postconsultation processes (eg, payment and medication delivery), which are particularly salient in platform-based telemedicine services. Accordingly, the objectives of this study were to (1) develop the TSEQ for Chinese outpatient telemedicine services; (2) evaluate its underlying factor structure using exploratory and confirmatory factor analyses; and (3) assess its reliability and validity, including internal consistency and convergent validity.

## Methods

### Data Collection and Sample

Our research focuses on users of hospital apps at Peking Union Medical College Hospital, a leading medical institution renowned for its comprehensive health care services and advanced medical technology. Data were collected through a web-based questionnaire from July 1, 2021, to August 31, 2021. Eligible participants were adults (aged ≥18 years) who completed a telemedicine consultation and provided informed consent. We excluded responses with substantial missing data, implausibly short completion times, or duplicate submissions where applicable. Convenience sampling was used, which is common in psychometric validation studies. In this study, patients who fully completed the TSEQ questionnaire were included (N=3337). The survey window was selected because telemedicine services at the study hospital had reached stable operational routines and sufficient user volume for psychometric evaluation. As this study focuses on instrument development and validation, the 2021 dataset provides an appropriate foundation to examine the factor structure and reliability and validity of the TSEQ.

### Ethical Considerations

A web-based informed consent was obtained before the survey via WeChat. Participants were allowed to withdraw from the survey at any time. All interviews of the survey were kept confidential and anonymous. This study was approved by the Peking Union Medical College Hospital Ethics Committee (S-k1545). Responses were collected anonymously (or deidentified), and no directly identifiable personal information was stored. Data were stored on secure servers with restricted access limited to the research team. Participants did not receive any compensation for participation in this study.

### Measures and Scale Development

The TSEQ was adapted from the Chinese Patient Experience Questionnaire (CPEQ; refer to Multimedia Appendix 1 for details) [15]. The CPEQ consists of 2 sections: part A includes 9 questions that collect basic demographic information of the participants, such as age, sex, employment status, and type of insurance and part B comprises 22 questions across 5 dimensions and is designed to comprehensively evaluate patient experience. On the basis of expert discussions on the CPEQ, we developed a 15-item questionnaire for telemedicine service experience, focusing on self-efficacy, information guidance, and humanistic care. Every question was assessed using a 5-point Likert scale (5=strongly agree, 4=agree, 3=unsure, 2=disagree, and 1=strongly disagree). The newly formed TSEQ is presented in Multimedia Appendix 2.

The validation and reliability of the TSEQ are the cornerstones of our research’s second objective. We assessed the instrument’s internal consistency and test-retest reliability, alongside its validity, to ensure it measures the intended constructs. An open-ended question was included at the end of the questionnaire to capture valuable, unstructured feedback from the respondents.

### Statistical Analysis

Descriptive statistics were used to summarize and describe the demographic characteristics of the participants, such as age, sex, occupation, and education. Categorical variables (eg, sex, occupation, and education) were presented as numbers (percentages), and continuous variables (eg, age) were reported as mean (SD).

### Exploratory Factor Analysis

Exploratory factor analysis (EFA) was first performed to explore the number of factors and the factor loading of each item using maximum likelihood estimation, which is the default estimator in Mplus (version 7.0; Muthén & Muthén). Before the factor analysis, Bartlett test of sphericity was usually used to examine whether these variables were independent of each other and suitable for factor analysis. In this study, we computed 1-factor, 2-factor, 3-factor, and 4-factor measurement models using EFA. To identify the model structure, we used a series of indicators such as *χ*^2^, *df*, Tucker-Lewis index (TLI), comparative fit index (CFI), Akaike information criterion, Bayesian information criterion [1,16], standardized root mean square residual (SRMR), and root mean square error of approximation (RMSEA) to evaluate the goodness-of-fit statistics. Acceptable model fit was defined by the following criteria: TLI>0.90, CFI>0.90, SRMR<0.05, and RMSEA<0.08 [17-19].

During EFA, items were considered for removal if they exhibited low factor loadings (eg, <0.40), substantial cross-loadings, low commonalities, weak item-total correlations, or conceptual redundancy. The refined structure was then tested using confirmatory factor analysis (CFA), and model fit indices guided the final scale specification.

### Model Validation Using CFA

Following EFA, we conducted the CFA to verify whether the hypothesized structure from EFA was consistent and valid. CFA is a statistical technique used to evaluate the effectiveness of the measurement model and examine the relationship between measured variables and underlying latent constructs or factors. In this study, first-order factors were verified for the dimensions of telemedicine service experience, and a second-order model was established to explain the relationships among multiple first-order factors. We computed and compared the overall fit coefficients, *χ*^2^/*df*, RMSEA, CFI, TLI, and SRMR of CFA models to remove inappropriate items and confirm the ideal model results. Moreover, the model fit was evaluated using the standardized factor loadings and *R*^2^.

### Reliability and Validity

To assess the reliability of CFA-confirmed models, we further computed Cronbach α and split-half reliability coefficients (SRCs) for the total questionnaire, final questionnaire, service efficiency (F1), post-treatment (F2), information guidance (F3), and humanistic care (F4). With regard to the reliability of a research instrument, statistical theory has determined that Cronbach α should be >0.7 [20,21]. Correlation analysis was used to investigate the correlation between the first-order factors and Chinese patients’ overall satisfaction and willingness to recommend. Finally, a validity test was conducted to verify the validity of the newly proposed Chinese adaptation of the patient experience measurement instrument by estimating the factor loadings to examine the relationships between observed variables and latent constructs that we wanted to measure. Standard factor loadings >0.70 were considered the criterion for convergent validity [22].

## Results

### Sociodemographic Characteristics

Among the 3337 participants, the mean age was 45.3 (SD 17.8) years. The majority were female (n=2182, 65.4%), had completed a bachelor’s degree or higher education level (n=1827, 54.8%), paid using health insurance (n=2292, 68.7%), and were seeking medical care outside their local area (n=1372, 41.1%; Table 1).

Table 2 presents the performance scores for the 15 individual items of the TSEQ in this study. These items primarily focus on various aspects of hospital services, including waiting times, service convenience, the communication style of physicians, and the respect and privacy protection afforded to patients during their medical visits. The average scores for each item generally range between 4.2 and 4.6, indicating that most respondents are satisfied with the services provided by the hospital and that the scores demonstrate relatively high consistency. These findings support further development of the scale for the EFA and the CFA.

**Table 1.** Demographic characteristics of the participants (N=3337).

Variables	Participants
Age (years), mean (SD)	45.3 (17.8)
Sex, n (%)	
Male	1155 (34.6)
Female	2182 (65.4)
Occupation, n (%)	
Government and public institutions	881 (26.4)
State-owned enterprise	536 (16.1)
Nonpublic enterprise	815 (24.4)
Others	1105 (33.1)
Education, n (%)	
Bachelor’s degree or higher education level	1827 (54.8)
Others	1510 (45.2)
Registration, n (%)	
Online	1782 (53.4)
Others	1555 (46.6)
Payment, n (%)	
Self-pay	1045 (31.3)
Insurance	2292 (68.7)
Allopatry treatment^a^, n (%)	
Yes	1372 (41.1)
No	1965 (58.9)
Virtual physician visits, n (%)	
Yes	1024 (30.7)
No	2313 (69.3)

a Allopatry treatment refers to seeking outpatient care outside the patient’s usual place of residence or registration (ie, receiving care in a nonlocal setting).

**Table 2.** Performance scores for the 17 individual items of the Telemedicine Service Experience Questionnaire.

Question number	Item	Value, mean (SD)
Q1	The waiting time for on-site registration was acceptable.	4.2 (1.0)
Q2	The waiting time for consultation with physicians was acceptable.	4.5 (0.8)
Q3	The consultation length was acceptable.	4.5 (0.7)
Q4	The waiting time for a planned examination was acceptable.	4.5 (0.7)
Q5	The waiting time for payment was acceptable.	4.6 (0.6)
Q6	The waiting time for medicine delivery was acceptable.	4.6 (0.6)
Q7	It was convenient to access self-service information inquiry devices.	4.5 (0.7)
Q8	Medication instruction services provided by the hospital can meet needs.	4.5 (0.7)
Q9	Physicians discussed my condition and care patiently.	4.5 (0.8)
Q10	Physicians explained examination results to me patiently.	4.5 (0.8)
Q11	Physicians discussed treatment with me patiently.	4.5 (0.8)
Q12	I was treated with respect and dignity during this visit.	4.5 (0.8)
Q13	My privacy has been fully protected during this visit.	4.6 (0.6)
Q14	All the medical staff was kind to me during this visit.	4.5 (0.8)
Q15	I got help from medical staff as soon as I had problems.	4.5 (0.8)

### EFA Findings

The results of the EFA indicated that the Bartlett test of sphericity yielded a *χ*^2^ value of 785.966 (*df*=51; *P*<.001; Kaiser-Meyer-Olkin value=0.950; Table 3), suggesting that the dataset used was suitable for factor analysis. Through this analysis, we successfully established a 4-factor structure, with these factors collectively explaining 78.49% of the total variance (Multimedia Appendix 3). This indicates that the TSEQ tool exhibits good structural validity, accurately reflecting various aspects of patient experiences in remote health care services.

**Table 3.** Fit indices for the exploratory factor analysis model.

Model	*χ*^2^ (*df*)	Tucker-Lewis index	Comparative fit index	Akaike information criterion	Bayesian information criterion	Standardized root mean squared residual	Root mean squared error of approximation (90% CI)
One-factor	9637.302 (90)	0.754	0.789	77359.640	77634.715	0.086	0.178 (0.175-0.181)
Two-factor	2597.384 (76)	0.923	0.944	70347.720	70708.377	0.028	0.100 (0.096-0.103)
Three-factor	1262.718 (63)	0.956	0.974	69039.054	69479.177	0.021	0.076 (0.072-0.079)
*Four-factor^a^*	*785.966 (51)*	*0.967*	*0.984*	*68586.302*	*69099.780*	*0.016*	*0.066 (0.062-0.070)*

a Italicized text indicates the selected final model.

Of the 15 items, 14 loaded meaningfully onto 1 of the 4 factors, with factor loadings ranging from 0.38 to 0.89. However, one item (item 12: “Physicians discussed my condition and care patiently”) had cross-loadings on factors 2 and 4. Despite this, considering the importance of this item, we did not remove it in the EFA stage. The EFA resulted in a 4-factor solution that explained 79.00% of the variance (Table 4).

**Table 4.** Results of the exploratory factor loadings matrix.

Item number	Item	Factor loading			
		Factor 1	Factor 2	Factor 3	Factor 4
Q1	The waiting time for on-site registration was acceptable.	*0.689* ^a^	0.038	−0.147	0.018
Q2	The waiting time for consultation with physicians was acceptable.	*0.670*	0.162	−0.053	0.037
Q3	The consultation length was acceptable.	*0.707*	−0.033	0.153	0.004
Q4	The waiting time for a planned examination was acceptable.	*0.744*	−0.019	0.102	0.002
Q5	The waiting time for payment was acceptable.	0.279	−0.024	*0.517*	0.092
Q6	The waiting time for medicine delivery was acceptable.	0.021	0.065	*0.887*	0.006
Q7	It was convenient to make an appointment for a planned examination.	*0.607*	0.021	0.083	0.052
Q8	Medication instruction services provided by the hospital can meet needs.	*0.521*	0.180	0.221	−0.026
Q9	Physicians discussed my condition and care patiently.	0.005	*0.875*	0.001	0.069
Q10	Physicians explained examination results to me patiently.	0.072	*0.928*	0.000	−0.032
Q11	Physicians discussed treatment with me patiently.	−0.011	*0.902*	0.030	0.055
Q12	I was treated with respect and dignity during this visit.	−0.001	*0.444*	0.000	*0.523*
Q13	My privacy has been fully protected during this visit.	0.273	0.186	0.048	*0.382*
Q14	All the medical staff was kind to me during this visit.	−0.001	0.054	0.033	*0.860*
Q15	I got help from medical staff as soon as I had problems.	0.266	−0.025	−0.017	*0.662*

a Italicized factor loadings indicate the highest loading for each item and were used to assign the item to the corresponding factor.

### CFA Findings

In the CFA, it was observed that the modification index for item 12 was excessively high across the 4 factors (>200; Multimedia Appendix 4). After its removal, the model fit improved in the CFA model. Therefore, the final model excluded item 12 (Table 5).

Given the 4 factors derived from EFA, an interpretation focusing on items with higher factor loadings for each factor, and their comparison to the TSEQ, was performed by the panel (Table 6). Factor 1 was labeled “Service Efficiency,” as it consisted of 6 items dealing with the convenience of telemedicine services. The second factor, consisting of 2 items, was called “Post-treatment,” as the content involves the time spent on medication dispensing and payment processes. Factor 3 comprised 3 items that captured information-related elements of telemedicine services; therefore, the title of this factor was “Information Guidance.” Finally, the 3 items in factor 4, which involve the physician’s’ attitudes toward patients, were labeled “Humanistic Care.”

**Table 5.** Overall model fit coefficients for the confirmatory factor analysis model.

Model fit index	Ideal standard	Common standard	Model results	Results after excluding item 12	Second-order model	Conclusion (first-order model)
*χ*^2^/*df*^a^	<3	<10	23.33	18.70	30.30	Model not ideal
Root mean square error of approximation	<0.08	<0.1	0.082	0.073	0.094	Model ideal
Comparative fit index	>0.9	>0.8	0.959	0.968	0.946	Model ideal
Tucker-Lewis index	>0.9	>0.8	0.948	0.960	0.933	Model ideal
Standardized root mean square residual	<0.08	<0.1	0.035	0.030	0.047	Model ideal

a *χ*2/df: the ratio of *χ*2 to df.

**Table 6.** Results of confirmatory factor analysis of the Telemedicine Service Experience Questionnaire scales.

First-order factor and indicators	Standardized factor loading^a^	*R* ^2^
Service efficiency (F1)		
Q1^b^	0.599	0.359
Q2	0.763	0.582
Q3	0.795	0.632
Q4	0.798	0.636
Q7	0.729	0.531
Q8	0.812	0.660
Post-treatment (F2)		
Q5	0.836	0.699
Q6	0.867	0.752
Information guidance (F3)		
Q9	0.935	0.873
Q10	0.951	0.904
Q11	0.953	0.909
Humanistic care (F4)		
Q13	0.808	0.652
Q14	0.879	0.772
Q15	0.846	0.716
Second-order model and indicators		
Service efficiency (F1)^c^	0.936	0.876
Post-treatment (F2)	0.826	0.683
Information guidance (F3)	0.820	0.672
Humanistic care (F4)	0.940	0.883

a The *P* values for the standardized factor loadings are all <.001.

b Q1-Q15 denote questionnaire items, the full wording of which is provided in Table 4.

c F1-F4 denote the 4 factors of the second-order model.

### Internal Consistency and Validity

As presented in Table 7, the final instrument obtained a Cronbach α coefficient of 0.946 and an SRC value of 0.872 for the total sample. The 4 factors—service efficiency, post-treatment, information guidance, and humanistic care—each obtained Cronbach α coefficients ranging from 0.840 to 0.962, and their SRC values were 0.877 (6 items), 0.840(2 items), 0.965 (3 items), and 0.892 (3 items), respectively. These results demonstrate that the TSEQ has good internal consistency.

The correlation coefficients between overall satisfaction and the 4 factors—service efficiency, post-treatment, information guidance, and humanistic care—were 0.941, 0.781, 0.857, and 0.901, respectively. All the correlations were statistically significant at the .01 level and demonstrate good internal validity. Moreover, as the average variances extracted values were >0.5 and the composite reliability values were >0.7, the TSEQ scale demonstrated high convergent validity (Table 7).

**Table 7.** The evidence for the reliability and validity of the Telemedicine Service Experience Questionnaire (TSEQ) scale and 4 factors.

Variable	Correlation^a^				Internal consistency		Convergent validity	
	TSEQ	Service efficiency (F1)	Post-treatment (F2)	Information guidance (F3)	Cronbach α	Split-half reliability coefficients	Average variances extracted	Composite reliability
TSEQ (14-items, mean)	—^b^	—	—	—	0.946	0.872	—	—
Service efficiency (F1)	0.941	—	—	—	0.876	0.876	0.567	0.886
Post-treatment (F2)	0.781	0.805	—	—	0.840	0.840	0.725	0.841
Information guidance (F3)	0.857	0.667	0.520	—	0.962	0.962	0.896	0.962
Humanistic care (F4)	0.901	0.760	0.624	0.788	0.876	0.876	0.714	0.882

a All Pearson correlation coefficients were statistically significant (*P*<.001).

b Not applicable.

### TSEQ Scoring

The TSEQ measures each item on a 5-point Likert-type scale, ranging from 1 (“strongly disagree”) to 5 (“strongly agree”). Calculating a total score involves summing the response scores for all 14 items of the TSEQ. The total score ranges from 14 to 70, with higher scores indicating higher levels of telemedicine service experience. The Chinese version of the questionnaire is shown in Multimedia Appendix 5.

## Discussion

### Principal Findings

This study developed and validated the TSEQ, an instrument for measuring telemedicine service experience in Chinese outpatients, based on the CPEQ [15]. In this study, EFA produced a revised 4-factor solution for the TSEQ, leading to modifications in the 5 domains of the CPEQ. Telemedicine services offer convenient medical solutions and effectively tackle the issue of uneven distribution of medical resources, especially in remote and rural areas [23,24]. The global COVID-19 pandemic has recently accelerated the adoption and expansion of telemedicine [25,26]. Importantly, our study does not aim to re-establish the well-known advantage that telemedicine reduces geographic barriers. Instead, it contributes a validated, telemedicine-specific patient experience instrument and highlights which service-process dimensions matter in Chinese outpatient telemedicine workflows. In particular, the emergence of a distinct “post-treatment” domain (eg, payment timing and medication delivery) reflects operational features that are often underrepresented in traditional in-person satisfaction tools and provides actionable targets for quality improvement. Our findings contribute to global efforts to standardize patient-reported evaluation of telemedicine quality. Although the TSEQ was developed for Chinese outpatient workflows, its domain-based structure may inform telemedicine service monitoring in other settings, subject to appropriate cross-cultural adaptation and validation.

In this analysis, the final instrument comprises 4 factors with 14 items, designed to evaluate patient satisfaction with telemedicine services and identify areas for service improvement. The EFA supported the viability of a 4-factor model. Compared to previous studies, the EFA uniquely identified “payment timing (item 5)” and “medicine delivery (item 6)” as a new single factor, while grouping “It was convenient to make an appointment for a planned examination (item 7)” and “Medication instruction services provided by the hospital can meet needs (item 8)” under the first factor, service efficiency. Notably, the item “I was treated with respect and dignity during this visit (item 12)” was originally categorized under “Humanistic Care” in the CPEQ framework but was removed in this study. This change may be attributed to the fact that patients using telemedicine services are more focused on the quality and efficiency of the service and may not experience significant nonverbal communication cues such as eye contact, body language, and tone of voice, which are typically present during face-to-face interactions with health care professionals [24,27].

Additionally, although “privacy and security risks (item 13)” showed lower factor loadings in the EFA, these aspects were still included in the TSEQ due to their critical importance in the telemedicine context [28,29], where patient privacy and security are paramount. Telemedicine also introduces challenges, including technology barriers, digital literacy gaps, privacy or security concerns, and constrained clinician-patient communication. By quantifying patient experience across distinct service domains, the TSEQ can help hospital identify where telemedicine workflows fail to meet expectations and prioritize targeted improvements.

Furthermore, CFA validated the 4-factor structure of the questionnaire. According to the pre-established CFI, and RMSEA thresholds, the second-order model can be considered acceptable. Only the *χ*^2^ test revealed a bad fit for the second-order model, likely due to the large sample size [30]. All standardized factor loadings were statistically significant (all *P*<.001) and ranged from 0.729 to 0.953. On the basis of the CPEQ [15], our study has introduced a second factor, defined as “post-treatment,” further demonstrating that the efficiency of posttelemedicine services also affects overall patient satisfaction. Analysis of the internal consistency of the Chinese version of the TSEQ showed that the Cronbach α of the entire scale was 0.95, indicating excellent scale reliability. All scales demonstrated good internal consistency, with Cronbach α of >0.80, indicating moderate to good test-retest reliability [30,31]. Moreover, CFA revealed strong correlations among the 4 factors, and further exploration of the relationships among the 4 factors confirmed satisfactory convergent validity based on the average variances extracted and composite reliability values [32,33].

The majority of participants in our study were female (2182/3337, 65.4%) and had completed bachelor’s degree or higher education level (n=1827, 54.8%). Nearly half (n=1372, 41.1%) of the participants were seeking medical care outside their local area. The composition of our study population underscores the broad relevance of our findings. This diversity is essential for understanding the multifaceted patient experiences within telehealth services, emphasizing the need for inclusive and accessible health care solutions that cater to varied demographic segments [30,33].

The TSEQ offers actionable value for routine quality monitoring and continuous improvement in outpatient telemedicine services. In practice, health care professionals and platform administrators can integrate the TSEQ as a brief postvisit feedback tool embedded within the telemedicine workflow (eg, immediately after consultation or after completion of postconsultation steps). Domain-level scores can be visualized in a dashboard to identify specific bottlenecks and track trends over time, enabling a closed improvement loop of “measure-diagnose-intervene-reassess.” For example, low scores in interaction-related domains may prompt targeted clinician training (eg, communication clarity and shared decision-making in remote settings), whereas low scores in the post-treatment domain can inform workflow redesign for payment, prescription processing, and medication delivery or collection. The TSEQ can also support benchmarking across departments, specialties, or service lines to prioritize resource allocation and evaluate the impact of operational changes. At the policy level, aggregated TSEQ indicators may complement use metrics by capturing patient-centered service quality; helping health systems move from “telemedicine adoption” to “telemedicine performance”; and guiding standards for service transparency, privacy protection, and equitable access for digitally disadvantaged groups.

### Limitations

Several limitations should be acknowledged. First, this study relied on self-reported survey responses collected immediately after telemedicine use, which may be subject to social desirability bias, recall bias, and common method variance. Second, participants were recruited using a convenience sampling approach from a single hospital setting, which may limit generalizability to other regions, health care tiers, and telemedicine platforms, especially where service workflows, payment pathways, and medication dispensing or delivery arrangements differ. Third, the sample characteristics may not fully represent telemedicine users with limited digital access or lower digital literacy; therefore, our estimates of service experience may be upwardly biased for populations facing stronger technology barriers. Fourth, data were collected during mid-2021, when telemedicine use and patient expectations were shaped by the COVID-19 pandemic context; experience patterns may shift in postpandemic periods when patients have broader choices between in-person and remote visits. Fifth, the cross-sectional design precludes assessment of temporal stability (eg, test-retest reliability), responsiveness to service improvements, and longitudinal predictive validity. Finally, although we established overall psychometric properties, formal measurement invariance and differential item functioning across key subgroups (eg, age, sex, education, allopatry treatment, and previous telemedicine experience) were not the primary focus of this manuscript and warrant further validation in multisite and postpandemic samples.

### Conclusions

In conclusion, this study developed and psychometrically validated the TSEQ for Chinese outpatients. The final instrument demonstrated a stable multidomain structure, indicating that it can be used to capture key dimensions of patients’ telemedicine service experience in routine outpatient care. By providing domain-specific scores, the TSEQ enables health care institutions and platform administrators to identify actionable targets for quality improvement, particularly in end-to-end workflow components that are salient in telemedicine settings (eg, communication processes and postconsultation services). Future research should validate the TSEQ in multisite and postpandemic samples, assess test-retest reliability and responsiveness to service changes, and examine measurement invariance or differential item functioning across key subgroups to support broader implementation and benchmarking.

## Supplementary material

10.2196/60551 Multimedia Appendix 1 Original version of the Chinese Patient Experience Questionnaire.

10.2196/60551 Multimedia Appendix 2 Newly formed version of the Telemedicine Service Experience Questionnaire.

10.2196/60551 Multimedia Appendix 3 Four-factor analysis.

10.2196/60551 Multimedia Appendix 4 Modification index.

10.2196/60551 Multimedia Appendix 5 Chinese version of the Telemedicine Service Experience Questionnaire.

10.2196/60551 Checklist 1 STROBE checklist.

## Abbreviations

CFA: confirmatory factor analysis
CFI: comparative fit index
CPEQ: Chinese Patient Experience Questionnaire
EFA: exploratory factor analysis
RMSEA: root mean square error of approximation
SRC: split-half reliability coefficient
SRMR: standardized root mean square residual
TLI: Tucker-Lewis index
TSEQ: Telemedicine Service Experience Questionnaire
