# Supplementary material for: Telemedicine Service Experience Questionnaire for Chinese Outpatients: Development and Validation Study
Source: JMIR Hum Factors. 2026 May 21;13:e60551. doi: 10.2196/60551 (PMC13193669; doi:10.2196/60551)
Supplement: Multimedia Appendix 2 [file humanfactors-v13-e60551-s002.docx]

**Multimedia Appendix 2** **The newly formed of the Telemedicine Service Experience Questionnaire**

| **Part A: patient demographic information** | | | | |
| --- | --- | --- | --- | --- |
| No. | Questions | Options | |  |
| S1 | Reservation or not? | 1 Reservation 2 Walk in | |  |
| No. | Patient Demographic information | Options | |  |
| S2 | Gender | 1 Male 2 Female | |  |
| S3 | Age | 1.18 to 24 2. 25 to 34 3. 35 to 44 4. 45 to 54 5. 55 to 64 6. 65 to 74 7.75 to 79 8. 80 to 84 9. 85above | |  |
| S4 | Education | 1 Graduate above 2 Batcheler degree 3 professional training 4 high school 5 middle school 6 primary school and below | |  |
| S5 | Occupation | 1 Government & Public Institutions 2 State Owned Enterprise 3 Non-Public Enterprise 4 Farmer 5 FreeLancer 6 Retired 7 no job 8 others | |  |
| S6 | Payment | 1 self-pay 2 commercial insurance 3 public insurance 4 others | |  |
| S7 | Specialist | 1Inner Medicine 2 Surgical Dept. 3 Gynecology Dept. 4 Obstetrics Dept. 5 Pediatrics Dept. 6 Traditional Chinese Medicine 7 ENT Dept. 8 Stemmatological Dept. 9 Ophthalmology Dept. 10 Dept. of plastic surgery 11 Dermatology Dept. 12 others | |  |
| S8 | Beijing patients or outside Beijing | 1 Live in Beijing 2 Live outside Beijing | |  |
| S9 | Service experienced | 1 Outpatients service 2 Digital Service 3 Outpatients and digital services | |  |
| **Part B: Experience Questionnaire** | | | | |
| Pick up one option based on your recent experience of our service (choose 0 if not experienced) | | | | |
| No. | Patients Experiences Questions | | Options | N/A |
| Q1 | The waiting time for on-site registration was acceptable | | 5 strongly agree 4 agree 3 unsure 2 disagree 1strongly disagree | 0 |
| Q2 | The waiting time for consultation with doctors was acceptable | | 5 strongly agree 4 agree 3 unsure 2 disagree 1strongly disagree | 0 |
| Q3 | The consultation length was acceptable | | 5 strongly agree 4 agree 3 unsure 2 disagree 1strongly disagree | 0 |
| Q4 | The waiting time for a planned examination was acceptable | | 5 strongly agree 4 agree 3 unsure 2 disagree 1strongly disagree | 0 |
| Q5 | The waiting time for payment was acceptable | | 5 strongly agree 4 agree 3 unsure 2 disagree 1strongly disagree | 0 |
| Q6 | The waiting time for medicine delivery was acceptable. | | 5 strongly agree 4 agree 3 unsure 2 disagree 1strongly disagree | 0 |
| Q7 | It was convenient to make an appointment for a planned examination | | 5 strongly agree 4 agree 3 unsure 2 disagree 1strongly disagree | 0 |
| Q8 | Medication instruction services provided by the hospital can meet needs | | 5 strongly agree 4 agree 3 unsure 2 disagree 1strongly disagree | 0 |
| Q9 | Doctors discussed my condition and care patiently | | 5 strongly agree 4 agree 3 unsure 2 disagree 1strongly disagree | 0 |
| Q10 | Doctors explained examination results to me patiently | | 5 strongly agree 4 agree 3 unsure 2 disagree 1strongly disagree | 0 |
| Q11 | Doctors discussed treatment with me patiently | | 5 strongly agree 4 agree 3 unsure 2 disagree 1strongly disagree | 0 |
| Q12 | I was treated with respect and dignity during this visit | | 5 strongly agree 4 agree 3 unsure 2 disagree 1strongly disagree | 0 |
| Q13 | My privacy has been fully protected during this visit | | 5 strongly agree 4 agree 3 unsure 2 disagree 1strongly disagree | 0 |
| Q14 | All the medical staff was kind to me during this visit | | 5 strongly agree 4 agree 3 unsure 2 disagree 1strongly disagree | 0 |
| Q15 | I got help from medical staff as soon as I had problems | | 5 strongly agree 4 agree 3 unsure 2 disagree 1strongly disagree | 0 |
