# Supplementary material for: Telemedicine Service Experience Questionnaire for Chinese Outpatients: Development and Validation Study
Source: JMIR Hum Factors. 2026 May 21;13:e60551. doi: 10.2196/60551 (PMC13193669; doi:10.2196/60551)
Supplement: Multimedia Appendix 3 [file humanfactors-v13-e60551-s003.docx]

**Multimedia Appendix 3 Factor Analysis**

|  |  | Initial Eigenvalues |  |
| --- | --- | --- | --- |
| Factors | Sum | % of Variance | Cumulative % |
| 1 | 10.59 | 62.27 | 62.27 |
| 2 | 1.47 | 8.64 | 70.91 |
| 3 | 0.73 | 4.28 | 75.19 |
| **4** | **0.56** | **3.30** | **78.49** |
| 5 | 0.53 | 3.11 | 81.60 |
| 6 | 0.45 | 2.65 | 84.25 |
| 7 | 0.39 | 2.31 | 86.55 |
| 8 | 0.38 | 2.26 | 88.82 |
| 9 | 0.33 | 1.95 | 90.77 |
| 10 | 0.30 | 1.77 | 92.53 |
| 11 | 0.26 | 1.56 | 94.09 |
| 12 | 0.26 | 1.51 | 95.60 |
| 13 | 0.23 | 1.33 | 96.92 |
| 14 | 0.17 | 1.01 | 97.93 |
| 15 | 0.15 | 0.88 | 98.81 |
| 16 | 0.11 | 0.66 | 99.47 |
| 17 | 0.09 | 0.53 | 100.00 |

Note：Extraction Method (Principal Component Analysis).
