# Supplementary material for: Telemedicine Service Experience Questionnaire for Chinese Outpatients: Development and Validation Study
Source: JMIR Hum Factors. 2026 May 21;13:e60551. doi: 10.2196/60551 (PMC13193669; doi:10.2196/60551)
Supplement: Multimedia Appendix 4 [file humanfactors-v13-e60551-s004.docx]

**Multimedia Appendix 4 The modification index**

| Factors |  | Items | M.I. | E.P.C. | Std E.P.C. | E.P.StdYX E.P.C.C. |
| --- | --- | --- | --- | --- | --- | --- |
| F1 | BY | B5 | 99.711 | 0.611 | 0.373 | 0.596 |
| F1 | BY | B6 | 271.938 | 0.99 | 0.605 | 0.976 |
| F1 | BY | B12 | 1890.099 | 0.988 | 0.603 | 0.784 |
| F1 | BY | B13 | 72.135 | 0.337 | 0.206 | 0.328 |
| F1 | BY | B1 | 188.073 | -0.713 | -0.435 | -0.543 |
| F2 | BY | B3 | 10.262 | -0.176 | -0.093 | -0.092 |
| F2 | BY | B4 | 93.418 | 0.307 | 0.162 | 0.234 |
| F2 | BY | B7 | 48.104 | 0.23 | 0.121 | 0.167 |
| F2 | BY | B8 | 31.27 | 0.198 | 0.104 | 0.145 |
| F2 | BY | B9 | 121.671 | 0.355 | 0.187 | 0.26 |
| F2 | BY | B12 | 13.892 | -0.066 | -0.035 | -0.043 |
| F2 | BY | B13 | 1389.687 | 1.01 | 0.531 | 0.691 |
| F2 | BY | B14 | 12.847 | 0.105 | 0.055 | 0.088 |
| F2 | BY | B15 | 101.019 | -0.355 | -0.187 | -0.233 |
| F2 | BY | B2 | 36.274 | -0.211 | -0.111 | -0.141 |
| F3 | BY | B3 | 55.355 | 0.163 | 0.123 | 0.158 |
| F3 | BY | B4 | 111.054 | -0.201 | -0.152 | -0.22 |
| F3 | BY | B5 | 84.276 | -0.183 | -0.138 | -0.19 |
| F3 | BY | B6 | 64.869 | -0.158 | -0.119 | -0.19 |
| F3 | BY | B7 | 63.144 | -0.152 | -0.114 | -0.185 |
| F3 | BY | B12 | 15.429 | -0.083 | -0.063 | -0.088 |
| F3 | BY | B13 | 2475.319 | 0.894 | 0.674 | 0.877 |
| F3 | BY | B14 | 86.205 | 0.164 | 0.123 | 0.197 |
| F3 | BY | B2 | 250.084 | 0.339 | 0.255 | 0.319 |
| F4 | BY | B3 | 24.018 | 0.283 | 0.143 | 0.184 |
| F4 | BY | B4 | 45.238 | -0.341 | -0.172 | -0.25 |
| F4 | BY | B5 | 30.017 | -0.291 | -0.147 | -0.203 |
| F4 | BY | B6 | 10.421 | -0.254 | -0.129 | -0.206 |
| F4 | BY | B12 | 95.885 | -0.756 | -0.383 | -0.618 |

Note: M.I., modification index;
