# Supplementary material for: Telemedicine Service Experience Questionnaire for Chinese Outpatients: Development and Validation Study
Source: JMIR Hum Factors. 2026 May 21;13:e60551. doi: 10.2196/60551 (PMC13193669; doi:10.2196/60551)
Supplement: Multimedia Appendix 5 [file humanfactors-v13-e60551-s005.docx]

**Multimedia Appendix 5** **The Chinses version of the TSEQ**

| 根据您最近的服务经验选择一个选项（如果没有经验，请选择0） | | | |
| --- | --- | --- | --- |
| No. | 远程医疗就诊体验满意度 | 选项 | N/A |
| Q1 | 我对挂号流程便利性满意 | 5 非常同意 4 同意 3 一般 2 不同意 1 非常不同意 | 0 |
| Q2 | 我对候诊过程满意 | 5 非常同意 4 同意 3 一般 2 不同意 1 非常不同意 | 0 |
| Q3 | 我对检验(血尿化验等)过程满意 | 5 非常同意 4 同意 3 一般 2 不同意 1 非常不同意 | 0 |
| Q4 | 我对检查(影像学检查等)过程满意 | 5 非常同意 4 同意 3 一般 2 不同意 1 非常不同意 | 0 |
| Q5 | 我对缴费过程满意 | 5 非常同意 4 同意 3 一般 2 不同意 1 非常不同意 | 0 |
| Q6 | 我对取药过程满意 | 5 非常同意 4 同意 3 一般 2 不同意 1 非常不同意 | 0 |
| Q7 | 北京协和医院APP提供的自助信息查询服务方便易用 | 5 非常同意 4 同意 3 一般 2 不同意 1 非常不同意 | 0 |
| Q8 | 医院提供的用药指导服务方便易用 | 5 非常同意 4 同意 3 一般 2 不同意 1 非常不同意 | 0 |
| Q9 | 医生与我耐心沟通病情 | 5 非常同意 4 同意 3 一般 2 不同意 1 非常不同意 | 0 |
| Q10 | 医生向我详细解释检查、检验结果 | 5 非常同意 4 同意 3 一般 2 不同意 1 非常不同意 | 0 |
| Q11 | 医生对我耐心介绍治疗方案 | 5 非常同意 4 同意 3 一般 2 不同意 1 非常不同意 | 0 |
| Q12 | 诊疗过程中我的个人隐私得到了的保护 | 5 非常同意 4 同意 3 一般 2 不同意 1 非常不同意 | 0 |
| Q13 | 医院工作人员的态度和蔼 | 5 非常同意 4 同意 3 一般 2 不同意 1 非常不同意 | 0 |
| Q14 | 我遇到问题时能够及时得到医院工作人员的帮助 | 5 非常同意 4 同意 3 一般 2 不同意 1 非常不同意 | 0 |
